# Gender differences in objective and self-reported sleep

**DOI:** 10.1093/sleepadvances/zpag048

**Published:** 2026-04-27

**Authors:** Torbjörn Åkerstedt, Johanna Schwarz, Eva Lindberg, Jenny Theorell-Haglöw

**Affiliations:** Department of Clinical Neuroscience, Karolinska Institute, Stockholm, Sweden; Department of Psychology, Stockholm University, Stockholm, Sweden; Department of Clinical Neuroscience, Karolinska Institute, Stockholm, Sweden; Department of Psychology, Stockholm University, Stockholm, Sweden; Department of Medical Sciences, Respiratory, Allergy and Sleep Research, Uppsala University, Uppsala, Sweden; Department of Medical Sciences, Respiratory, Allergy and Sleep Research, Uppsala University, Uppsala, Sweden

**Keywords:** sex, polysomnography, age, self-reported sleep, PSG, awakenings

## Abstract

It is not clear whether gender differences in self-reported sleep reflect polysomnographical differences. We here investigated gender differences in polysomnography (PSG) variables and their association with rated sleep quality for recorded sleep. The participants were 238 women and 238 men who were recorded for a night with PSG (home recordings), and provided sleep quality ratings for the recorded night. Analyses of variance showed that women reported significantly lower sleep quality than men, but it showed significantly better PSG sleep (fewer awakenings per hour, lower N1%, longer total sleep time, higher sleep efficiency, and more N3%, among others). However, men underestimated their objective number of awakenings and had a shorter objective time awake per objective awakening (6.4 ± .6 vs 8.2 ± .6 minutes for women, *p* < .05). Men with short awakenings (<7.8 min per awakening) had a high self-reported sleep quality, in contrast to men with long awakenings or women regardless of the duration of awakenings. When men with short awakenings were excluded, self-reported sleep quality no longer differed between genders. Gender differences in PSG variables increased with age. In addition, better self-reported sleep quality was associated with “better” PSG values for both genders. In conclusion, women reported poorer sleep quality than men but showed better objective sleep. It is suggested that men’s better self-reported sleep is associated with an inability to perceive/remember short awakenings. The findings open a new view of gender differences in sleep, and indicate a need for experimental studies on gender differences in the perception of awakenings, their duration, and rated sleep quality.

**Statement of Significance**

The lack of correspondence between gender differences in objective and self-reported sleep seems paradoxical. The present study suggests that the lack of correspondence may be, at least partly, due to men’s inability to perceive short awakenings. This may lead to a new understanding of gender differences in sleep and their importance. Ascertaining the reasons for the male difficulty in perceiving short awakenings should be an important next step.

## Introduction

Self-reported sleep problems have a high prevalence in the population, 17.5% for insomnia and 26.6% for “subsyndromal insomnia” [1], and are linked to a number of diseases, including cardiovascular disease, Alzheimer’s disease, and diabetes [2]. The prevalence of insomnia is higher in women than in men [3]. Also, sleep problems in the population in general are higher in women than in men [4–6]. Women also more frequently seek treatment for insomnia—in the largest study of insomnia treatment, 71.5% of patients who had sought help were female [7]. It should be emphasized that the exact definition of “sleep problems” or “sleep quality” differs between studies, but the clinical definition of insomnia involves at least one of the following frequent difficulties: falling asleep, maintaining sleep, or sleeping through (early awakenings), plus reduced daytime functioning [8].

However, while self-reported sleep problems are more prevalent in women than in men, it is not clear to what extent objective sleep (as measured by polysomnography [PSG]) reflects this difference. A recent meta-analysis of gender differences (169 studies), focusing on nonclinical populations, showed rather few significant differences [9]. However, males had a shorter REM latency, a higher arousal index, and a higher apnea–hypopnea index (AHI) than women. The latter two suggest a lower quality of objective sleep in men, rather than in women, but major PSG variables, including total sleep time (TST), sleep efficiency, and number of awakenings, did not seem to differ. Nonetheless, meta-analyses involve compromises across studies, and the interstudy variability in age and exclusion criteria might make differences difficult to identify. The studies involved in the meta-analysis excluded individuals with sleep problems (including sleep apnea), use of sleep medication, and gender differences. A sample of the general population may give another impression of gender related PSG differences.

Results from individual studies paint a different picture, however. Bixler *et al*. found, in over 1300 randomly selected nonpatients, more stage 3 + 4 sleep (slow-wave sleep [SWS]), less N1%, and a higher sleep efficiency in women compared with men [10]. Traditionally, such values are considered to represent “better” sleep, together with other variables like short sleep latency and low number of awakenings. Luca *et al*., in another large study (*N* = 1147) [11], also found better objective sleep quality in women, that is, a longer TST, higher sleep efficiency, lower time awake after sleep onset (WASO), shorter SWS latency, and higher SWS%. However, the age range was limited to 35–75 years. In a study of our own (*N* = 60), we obtained similar results [12], with an age range of 20–75 years. Mitterling *et al.*, finally, found in 100 participants less N1% and more N2% in women, although an upper age limit of 60 years may have restricted the results [13]. The impression from these PSG studies of gender differences is that women objectively seem to sleep better than men. Whether this was reflected in self-reports of sleep quality for the measured nights was not investigated in any of the studies, unfortunately. Against the background of the arguments above, it would be interesting to investigate gender differences in PSG variables in a representative large sample of the population, without exclusion of poor sleepers, and also to investigate to what extent gender differences in objective sleep align with reported sleep quality during the same night.

To understand more about gender discrepancies in objective and self-reported sleep, one might also need to investigate the relation between self-reported and objective measures of quantitative sleep variables. This may give indications of self-reported over- or underestimates of objective sleep characteristics. Such variables include the ratio between self-reported and objective measures of sleep duration, number of awakenings, sleep latency, and time awake (WASO). Gender-related overestimation or underestimation of PSG measures might indicate reasons for gender-related differences in self-reported sleep quality between men and women. Another interesting additional variable is PSG-based time awake per awakening, since it appears that an awakening may not be remembered in the morning unless there has been 4–5 min of wakefulness involved [14]. Also here, gender differences may help explain gender differences in self-reported sleep quality.

Yet another factor that influences sleep, and which may have different gender effects in objective/self-reported sleep, is age. While sleep continuity variables and N3 decrease with age [9, 15], as do survey data of sleep quality [16], we know very little about gender differences. However, one study showed that, whereas PSG indicators of good sleep decreased faster in men than in women, the opposite was true for self-reported sleep complaints [17]. This was not included in the meta-analyses of Ohayon *et al.* and Boulos *et al.* [9, 15], but one study has investigated this, finding a significant age^*^gender interaction, indicating that women have a lower decrease in N3 than men with increasing age [12]. Bixler *et al.* showed that women have a lower N1% and higher N3% than men with increasing age [10], although their interaction was not formally tested. Thus, consideration of age may shed further light on gender differences in objective/self-reported sleep.

The purpose of the present study was to investigate gender differences and age^*^gender interactions in polysomnographic variables in a large group of men and women, and to compare these with reported sleep quality in the morning after awakening from the recorded sleep. The main questions were: do men and women differ in objective sleep (PSG) and rated sleep quality for specific recorded sleep? Are gender differences associated with age? Do men and women have the same association between PSG variables and the self-reported quality of the recorded sleep?

## Materials and methods

### Participants

**Figure 1:**
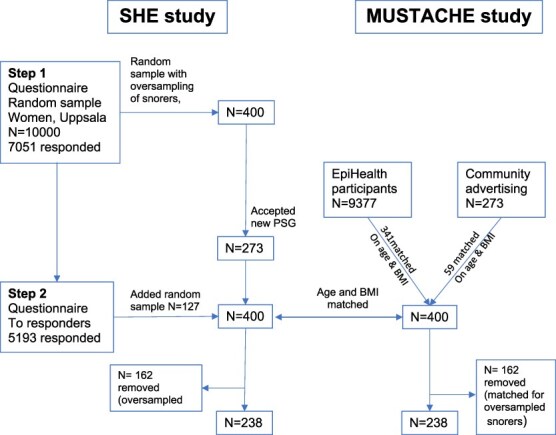
Flow chart for selection of participants in the SHE and Mustach studies.

The SHE (women only) study was based on a questionnaire sent to 10 000 women in Uppsala, Sweden, of whom 7051 responded (Figure 1) [18]. From these, a random sample (*n* = 130) plus a random sample of frequent snorers (*n* = 230) was selected (*N* = 400). The oversampling of snorers was intended for another study and was not used in the present one. All participated with a polysomnographic recording of sleep (and sleep ratings). Ten years later all the women who had responded to the first questionnaire were sent a new questionnaire. The previous participants who had performed a polysomnography were asked to participate again, and 273 accepted. An additional 127 women were randomly selected (no oversampling of snorers) from all the responders to the new questionnaire, making up a total of 400 women who were investigated during 2012–2014. Of these, 162 had been oversampled for frequent snoring at baseline and were removed, leaving *n*= 238, constituting a random sample. Figure 1 shows the study flow.

The MUSTACHE (“male”) study recruited male participants from the community-based study Epi-Health (*N* = 9377), matched with the women in the SHE cohort by age and BMI [18]. If no match was found (*n* = 59), a matching man was recruited from the community by local announcements, resulting in a total sample of 400 participants. Because the matching included the 162 oversampled women, men matching these women were removed, resulting in *N* = 238. As with the women, the men were investigated with a whole night of PSG, questionnaires, and other measures, according to the same protocol as in the SHE study. Figure 1 shows the study flow in the MUSTACHE study and matching with the SHE study.

The Ethics Committee at Uppsala University, Uppsala, Sweden, approved the study protocol (approval numbers 2009/379 and 2016/029). Written informed consent was obtained from all the participants. The data that support the findings of this study are not publicly available due to privacy or ethical restrictions.

### PSG recording and ratings

Sleep was recorded in the home, using the Embla (Flaga, Iceland) portable solid-state sleep recorder. We used a standard electrode (silver/silver chloride) montage (C3, C4) referenced to the contralateral mastoids. Two sub-mental electrodes, as well as electrodes at the outer canthi of the eyes, were also applied. In accordance with AASM scoring, F4 was interpolated. One research nurse applied the electrodes, connected the equipment, and gave instructions in the early evening. Next morning, the equipment was retrieved by a research nurse. Diary information was used to establish lights out and lights on times.

Standard sleep scoring was performed according to the classification criteria of the American Academy of Sleep Medicine [19]. The main variables were TST, sleep latency, latency to N3, latency to REM, sleep efficiency, WASO (wake after sleep onset), number of awakenings per hour, and minutes and percentage (of TST) of stages N1, N2, N3, and REM. In addition, several new variables were constructed:

- Sleep duration subj/obj = ratio of reported sleep duration and TST from PSG recording.
- Awakenings subj/obj = ratio of number of reported awakenings in the sleep diary and the number of PSG-based awakenings. This variable was intended to reflect how sensitive self-reported awakenings were to objective awakenings.
- Sleep latency subj/obj = ratio of self-reported/objective sleep latency.
- Time awake subj/obj = ratio of self-reported/objective time awake after sleep onset.
- Time awake per awakening = ratio of objective total time awake (from sleep onset to offset) to PSG-determined number of awakenings.

Also, variables associated with obstructive sleep apnea (OSA) were scored. An apnea was defined as a cessation of airflow for at least 10 s, while a hypopnea was defined as at least 10 s of 50% reduced respiratory volume, together with at least a 3% desaturation or an arousal. The AHI was defined as the mean number of apneas and hypopneas per hour of sleep.

From the questionnaire several variables were used. This included “use of sleep medication” (“never” [1] to “very often” [5]), married/cohabiting (no/yes, 0/1), BMI (kg/m^2^), regular alcohol intake (1=never, 2=occasionally, 3=once a week, 4=several times a week), self-reported health (7 = low, 1 = high), snoring frequency (1 = low, 5 = high), always stressed (1 = never, 5 = always), and smoking (no/yes).

For sleep diary self-ratings in the morning after the recorded sleep, the Karolinska Sleep Diary was used [20, 21]. We selected the sleep quality index, which represents the mean of four sleep items similar to those used to identify insomnia [8]: difficulties falling asleep, restless sleep, early morning awakenings, and sleep quality (scored from 1- to 5, with higher values reflecting better sleep). The sleep quality index was also used as a fixed factor in the analyses of variance, with levels 1 and 2 combined since only 14 participants were found with the rating 1. Similarly, groups 4 and 5 were combined since only 12 participants were found with the rating 5. The resulting three levels ranged between scores of 1 and 2.99 (labeled “2” in graphs, *n* = 78), 3.0 and 3.99 (labeled “3.5,” *n* = 174), and 4.01–5 for high sleep quality (labeled “4.5,” *n* = 230).

### Statistical analysis

Only data collected in 2012–2014 were used in the analyses. Descriptives are presented as mean ± SD (or %) for background and other variables. The main analysis was an analysis of variance (ANOVA) with gender and age as fixed factors and the PSG variables and the sleep quality index as dependent variables. Age was divided into three categories to represent the age ranges: 29–49 years (*n* = 151), 50–64 years (*n* = 180), and 65–85 years (*n* = 147). The latter represents the normative retirement year in Sweden and may be an important influence on the time window available for sleep. However, it has been gradually extended during the last two decades. Analyses were adjusted for smoking (no/yes), use of alcohol (1–4), and use of sleep medication (1–5). Depending on the results of the ANOVA, explorative analyses were carried out for further understanding of gender differences in self-reported/objective sleep. Also, a second ANOVA was carried out, with the sleep quality index (mean of the three sleep quality items), gender, and PSG variables as dependent variables. Here, the continuous variable “age” was entered as a covariate, together with the covariates used in the previous analysis. All the analyses were carried out with IBM SPSS 29.0.

## Results

Table 1 shows the background data for men and women. Women were somewhat younger, were more frequently married, had a lower alcohol intake, had lower self-reported health, and reported less snoring, more stress, and more use of sleep medication.

**Table 1:** Background data for men and women. Results are presented as mean ± SD or *n*/%, and *p*-values. *N* = 476.

	**Women**	**Men**	***P* <**
***n***	238	238	
**Age (years)**	56.7 ± 11.7	57.6 ± 12.1	ns
**Married/cohabiting (yes), *n*/%**	161/67.6	239/60.6	.001
**BMI (kg/m** ^**2**^ **)**	25.6 ± 4.9	25.4 ± 4.2	ns
**Smoker now (yes)**	36/15.4	13/5.6	ns
**Alcohol, regular intake (3–4) (%)**	62/26.1	102/43.0	.001
**Self-reported health (7–1 [high])**	2.32 ± 0.16	2.06 ± 0.93	.01
**Snoring frequency (1–5 [high])**	2.62 ± 1.52	3.03 ± 1.50	.01
**Sleep medication (2–5 high), *n*/%**	47/19.7	17/7.1	.001
**Always stress (1–5 [always])**	1.96 ± 0.7	1.71 ± 0.6	.001

### Gender and age differences in sleep variables

**Figure 2:**
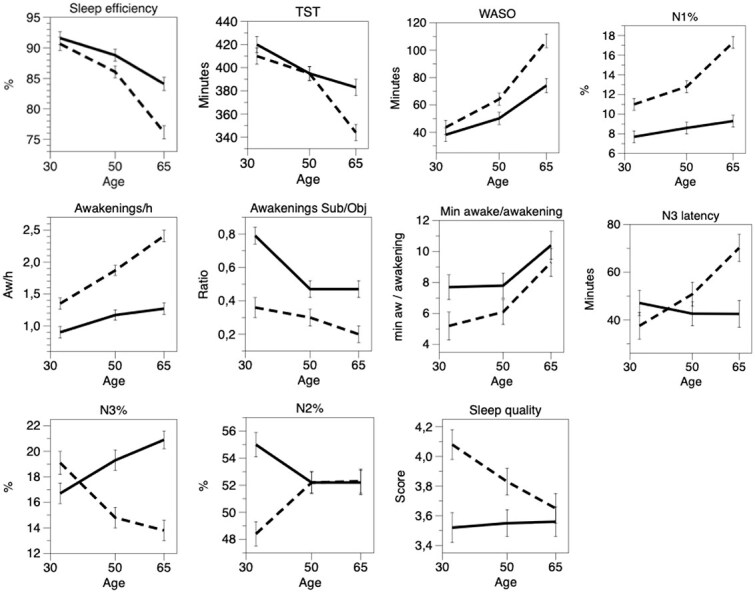
Age and gender versus PSG variables and ratios between self-reported and objective sleep variables. All the variables with significant effects for gender and/or with gender/age interaction are presented. Mean ± SE. Continuous line = women; dashed line = men. Mean ± SE.

Table 2 and Figure 2 show that women had significantly higher values for TST, sleep efficiency, N3%, N2%, self-reported/objective awakenings, REM latency, and time awake per awakening, while the values were significantly lower for WASO, N3 latency, awakenings per hour, and N1% (Figure 2). Figure 2 presents results for variables that show significant gender differences and/or gender/age interaction. The sleep quality index was significantly lower (worse) for women (Table 2 and Figure 2, last row). See also Supplementary Table S1 for means ± SD for variables that were not significant for gender or gender^*^age interaction in Table 2.

**Table 2:** Results from ANOVA with gender and age as fixed factors and PSG variables, as well as ratios between self-reported and objective variables, and the sleep quality index as dependent variables. Results are presented as *F*-ratios and *p*-values, adjusted for smoking, alcohol use, and use of hypnotics. *N* = 466 (some losses due to incomplete ratings).

	**Age *F*-ratio, *P***	**Gender *F*-ratio, *P***	A^*^G *F*-ratio, p	**Eta** ^**2**^
**TST**	29.6^***^	9.6^**^	4.7^*^	.12/.02/.02
**Sleep efficiency (%)**	54.7^***^	20.1^***^	5.6^**^	
**WASO (min)**	50.9^***^	18.5^***^	3.9^*^	.18/.04/.02
**Sleep latency**	4.4^*^	2.0	0.6	.02/.00/.00
**N3 latency (min)**	3.4^*^	4.0^*^	5.6^**^	.02/.01/.02
**REM latency (min)**	0.3	4.8^*^	0.4	.00/.01/.00
**AHI (/h)**	41.3^***^	0.0	0.4	.15/.00/.00
**N1%**	22.3^***^	110.2^***^	8.3^***^	.09/.19/.04
**N2%**	0.7	6.0^*^	11.4^***^	.00/.01/.05
**N3%**	0.0	20.8^***^	17.5^***^	.00/.04/.07
**REM%**	19.0^***^	0.5	1.6	.08/.00/.00
**Awakenings per hour**	31.5^***^	109.5^***^	7.4^**^	.12/.19/.03
**Time awake per awakening**	9.1^***^	5.9^**^	0.4	.04/.02/.00
**Sleep quality index**	1.8	14.8^***^	2.9	.00/03/.01
**Sleep duration subj/obj**	3.5^*^	2.2	0.5	.02/.00/.00
**No. awakenings subj/obj**	11.1^***^	44.8^***^	3.2^*^	.05/.10/.02
**Time awake subj/obj**	0.6	1.5	4.0^*^	.00/.00/.03
**Sleep latency, subj/obj**	0.2	1.7	0.1	.00/.00/.00

Abbreviations: AHI, apnea–hypopnea index; REM, rapid eye movement sleep; TST, total sleep time; WASO, time awake after sleep onset. ^*^p < .05, ^**^p < .01, ^***^p < .001.

Most PSG variables varied with age, except for REM latency, N2%, and N3%. TST, sleep efficiency and REM% decreased significantly with age, while all the other significant variables increased (Table 2 and Figure 2). Essentially, objective sleep quality deteriorated with age. For most of the significant PSG variables, also the age^*^gender interaction was significant, indicating increased gender differences, with more negative values for men, with higher age. The exception was self-reported/objective awakenings per hour, which decreased with age; men and women became more similar with increasing age.

**Figure 3:**
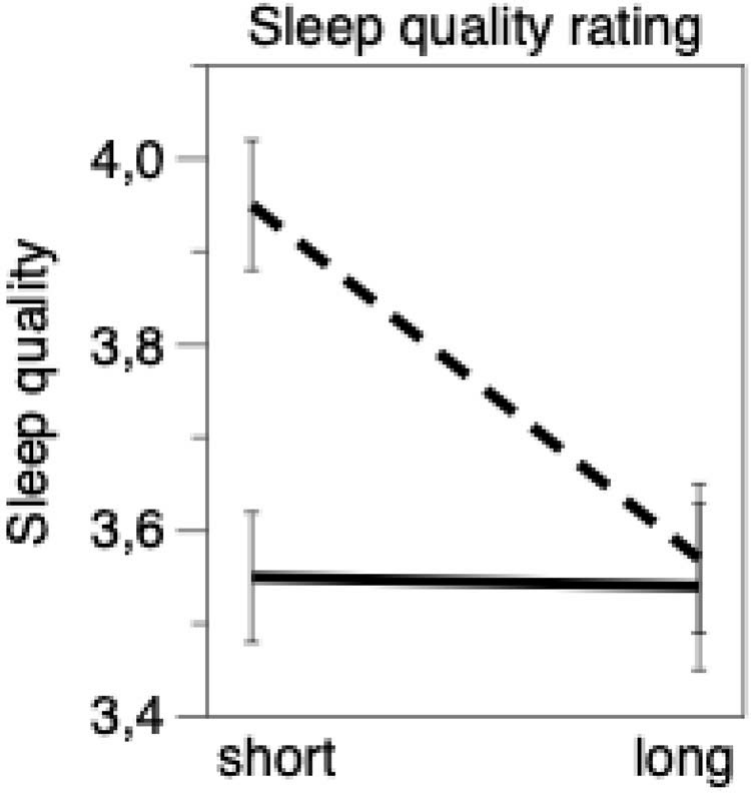
Results from ANOVA for short/long time awake per awakening (dichotomized at 7.8 min) and gender versus rated sleep quality for the same sleep. Continuous line = women; dashed line = men. Mean ± SE.

The only objective variable that seemed to indicate worse objective sleep for women was objective time awake per awakening. We, therefore, carried out an explorative analysis to investigate whether the observed gender difference in self-reported sleep quality would be influenced by wake time per awakening and gender group. We applied an analysis of variance with gender and time awake per awakening (dichotomized as <7.8 min vs ≥7.8 min) as fixed factors, with the sleep quality index as a dependent variable. This dichotomization was set at the 66th percentile (2/3) of the value of the variable. The results yielded significant *F*-ratios for wake time per awakening (*F* = 5.2, *p* < .05), gender (*F* = 6.2, *p* < .05), and interaction (*F* = 4.8, *p* < .05). Figure 3 shows that for women, sleep quality was low for both short (*n* = 153) and long (*n* = 83) awakenings, while for men it was high for short awakenings (*n* = 168) and low for long awakenings (*n* = 69). Post hoc analyses showed a significant difference within the male group (*p* < .001) and between males and females for short time awake per awakening (*p* < .001). The difference was not significant within the female group (*p* > .05), or between males and females, for long awakenings.

To illustrate the impact of men with short awakenings on self-reported sleep quality, we removed these men and compared self-reported sleep quality between the remaining men and all the women in an ANOVA. This yielded *F* = 0.40 (ns) with mean ± SE of self-reported sleep quality = 3.51 ± .07 for women (*n* = 238) and 3.61 ± .04 for men *n* = 67), respectively.

### Analysis of the self-reported sleep quality index versus PSG and other variables

Apart from the direct comparison of PSG variables between genders, as well as between age groups, we also analyzed whether men and women would have a significant association between diary sleep quality and PSG (and other) variables. For this purpose, we applied an ANOVA using the diary sleep quality index (divided into three categories) and gender as fixed factors, with age as a covariate (in addition to the covariates in the analysis above).

**Figure 4:**
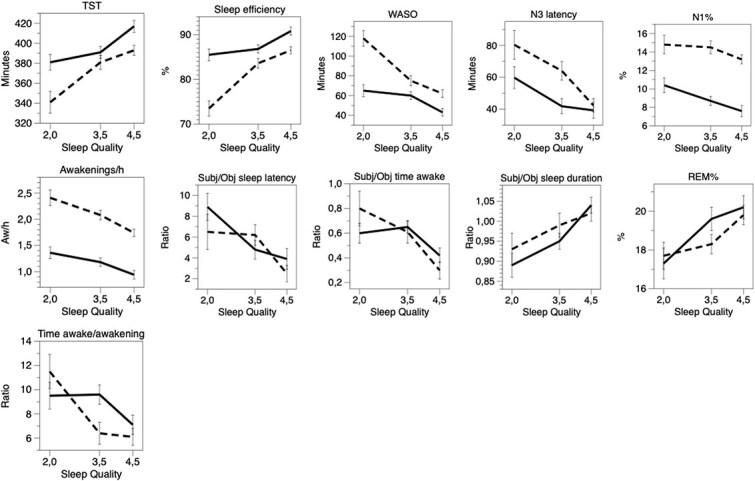
Results from ANOVA for self-reported sleep quality (three levels) versus PSG variables (with significant effects for sleep quality and gender). Continuous line = women; dashed line = men. Mean ± SE.

The results show that with higher-rated sleep quality, TST and sleep efficiency increased significantly, while WASO, N3 latency, N1%, and number of awakenings per hour decreased significantly. In addition, all the variables showed significant gender differences (Table 3 and Figure 4) Furthermore, time awake per awakening, self-reported/objective time awake, and self-reported/objective sleep latency decreased significantly, while self-reported/objective sleep duration and REM% increased significantly but showed no gender differences or interactions. The remainder of the variables did not show a significant association with sleep quality (results for mean ± SE presented in Supplementary Table S2.

**Table 3:** ANOVA with sleep quality index (three levels) and gender as fixed factors and PSG variables as dependent factors. Also included are self-reported and self-reported/objective variables. Results are presented as *F*-ratios and *p*-values. *N* = 450 (some losses occurred because of incomplete data).

	**Sleep quality *F*, *P***	**Gender *F*, *P***	**Interaction *F*, *P***	**Eta**
**TST (min)**	17.0^***^	17.5^***^	1.9	.07/.03/.01
**Sleep efficiency (%)**	29.9^***^	40.3^***^	6.4^**^	.12/.10/.03
**WASO**	25.4^***^	44.2^***^	5.3^**^	.10/.09/.02
**Sleep latency (min)**	0.5	0.7	0.4	.01/.00/.00
**N3 latency (min)**	11.1^***^	8.7^**^	1.7	.05/.02/.01
**REM latency**	4.1^*^	0.2	2.8	.01/.00/.00
**AHI (/h)**	1.3	0.0	0.0	.00/.00/.00
**N1%**	5.9^**^	74.4^***^	0.5	.02/.16/.00
**N2%**	0.8	2.0	0.2	.00/.00/.00
**N3%**	0.6	16.6^***^	0.1	.00/.04/.00
**REM%**	5.7^**^	0.9	0.5	.02/.00/.00
**Awakenings per hour**	15.7^***^	124.5^***^	0.7	.07/.22/.00
**Time awake per awakening**	7.7^***^	0.7	3.1^*^	.03/.00/.01
**Sleep duration, subj/obj**	20.3^***^	0.4	0.6	.11/.00/.00
**Awakenings, subj/obj**	0.2	29.3^***^	0.3	.00/.06/.00
**Time awake, subj/obj**	11.5^***^	0.4	1.4	.01/.00/.00
**Sleep latency, subj/obj**	7.6^***^	0.5	1.6	.04/.00/.01

Abbreviations: AHI, apnea–hypopnea index; REM, rapid eye movement sleep; TST, total sleep time; WASO, time awake after sleep onset. ^*^p < .05, ^**^p < .01, ^***^p < .001

## Discussion

The results show that, while self-reported sleep quality was lower in women than in men, most PSG variables showed better objective sleep in women. Thus, women obtained more TST, had a higher sleep efficiency, had fewer awakenings, had more N3%, etc. The difference increased with age. The only PSG variable that suggested worse objective sleep in women was time awake per awakening, which was higher in women. In addition, self-reported sleep quality for the recorded sleep was associated with longer TST, higher sleep efficiency, fewer awakenings, and other indicators of good objective sleep, but in most cases the intercept showed a gender difference, with women reporting worse sleep.

The better objective sleep in women (Table 2 and Figure 2) agrees with previous studies [10–13], but here we present a larger set of the traditional PSG variables. It shows that the lower number of awakenings per hour, WASO, and N1% in women, as well as their higher sleep efficiency and longer TST, indicates better sleep continuity for this group, that is, more sleep with less interruptions. Also, their higher N3% and shorter N3 latency should probably be seen as indications of good sleep, as well as possibly the shorter REM latency. These observations are in contrast to the more negative *ratings* of sleep quality in women. The rather paradoxical gender discrepancy does not seem to have been explicitly investigated before, although the better objective sleep of women has been documented in PSG studies [9–12], and the worse self-reported sleep quality in survey studies (of habitual sleep) [4–6].

Regarding age, many PSG variables showed a deterioration in sleep continuity and similar variables with higher age, which was expected from previous work [9, 15]. More importantly with respect to the purpose of the present study, gender differences increased with higher age for many PSG variables (Table 2; see also Figure 2). This was true, particularly, for N3%, but also for awakenings per hour, N1%, and WASO, with increasingly more negative values for men than for women. Very little comparable research is available, apart for two studies that show similar results [10, 12]. The reason for the gender discrepancy with increasing age seems worthy of further research, and certainly the dramatic differences in N3%.

When trying to understand the gender discrepancy in the present study, one may consider women’s significantly higher ratio for self-reported/objective number of awakenings. This was around 0.63 (see Figure 2), which is considerably closer to 1.0 (which would be the perfect estimate) than that of men (around 0.28). This suggests an inability of men to perceive awakenings, which could contribute to more positive sleep quality ratings in men. One may also consider men’s significantly shorter objective time awake per objective awakening (Figure 2) because a long time awake seems needed in order to remember an awakening [14]. Thus, one gets the impression that men, again, may have a lower ability to perceive objective awakenings than women.

The reasoning above was taken one step further through the exploratory analysis (Figure 3). It showed that women with short awakenings (<7.8 min per awakening), or long (≥7.8) awakenings, rated their sleep as being of similar, but modest, quality (Figure 3), as did men with long awakenings. However, men with short awakenings rated their sleep as significantly better than those three groups. When the latter group was excluded from the data set for illustrative purposes, the gender difference in sleep quality ratings was no longer significant. This supports the notion that men’s higher self-reported sleep quality, compared with that of women, may be, at least partly, due to men with short awakenings having difficulties perceiving or remembering awakenings. The reason for this difficulty is not known. Different AHI levels could be a possible contributor, but such a difference was not observed. The reason for men with short awakenings not perceiving them should be a very interesting topic for further work.

Despite the gender discrepancy discussed above, it should be emphasized that the combined gender groups showed significant associations between self-reported sleep quality and many objective variables, particularly sleep efficiency, WASO, TST, number of awakenings per hour, N3 latency, and time awake per awakening. High self-reported sleep quality was associated with good objective sleep quality. The results agree with previous work on self-reported sleep quality and specific recorded sleep [22–26]. One may note that a short N3 latency (see Figure 3) was associated with high sleep quality, whereas a high N3% was not.

In addition, Figure 4 and Table 3 show the logical finding that longer estimates of sleep duration and shorter estimates of time awake and sleep latency are linked to higher self-reported sleep quality. However, one should note that the correctness of the estimate seems irrelevant. For self-reported/objective number of awakenings, no similar associations were seen. This could perhaps be due to the large overall underestimation of the number of objective awakenings.

Since the interaction term for sleep quality and gender was not significant for most variables (Table 3), it appears that men and women had a similar association between self-reported sleep quality and PSG variables. This suggests that men and women perceive the quality of objective sleep in the same way. However, the main effects of gender showed that the association between self-reported sleep quality and, for example, number of awakenings (and sleep efficiency, etc.) for women operated on a lower level of sleep quality than that for men. We suggest that this difference in level of operation may be due to men with short and unperceived awakenings overestimating their sleep quality. This needs to be confirmed in further research, however.

The gender-related self-reported/objective mismatch should be seen against the background of the concept of “sleep state misperception” (or “paradoxical insomnia”), which has been an enigma to clinical sleep research for a long time [27]. Interestingly, sleep state misperception is interpreted as an inability to perceive that one is asleep [27], probably because of a high level of arousal. The present study suggests, however, that it may also include an inability to perceive that one is awake. Gender aspects of sleep state misperception do not seem to have been addressed before.

The strengths of the present study are its large size and full set of PSG variables, as well as the use of sleep quality ratings for the recorded sleep. Also, the introduction of wake minutes per awakening and the ratio of objective to self-reported awakenings introduced new possibilities of interpretation. Among the weaknesses may be the use of unsupervised home recordings in individuals following their normal life routines. This may have affected particularly objective sleep latency since self-reported lights out was used as a reference. Still, the home setting probably contributed to less stress and may have resulted in more natural sleep than a sleep laboratory setting. The exclusion of the oversampled snorers caused a loss of participants, but may have avoided confounding. Thus, differences in such variables did not contribute to the findings. This may be positive from the point of view of less confounding, but also negative, from the point of view of drawing conclusions from a representative sample. It should also be noted that the gender groups were matched on age and BMI. The latter probably reduced gender differences in PSG variables related to respiratory disturbances that are seen in an unselected population. On the other hand, the differences that were observed should have avoided confounding by BMI differences. One additional limitation may be that sensors for leg movements were not used in the study, which precluded the calculation of indices of such measures. This may have affected the gender differences in the number and duration of awakenings.

In conclusion, the present study revealed that a number of PSG variables show better sleep for women than for men, despite women reporting lower sleep quality for the same recorded sleep. It appears, however, that men with short awakenings have difficulties in perceiving them, and therefore rate their sleep as being of higher quality compared with other men (or women). This may contribute to the male group having higher ratings of sleep quality than women. Furthermore, self-reported sleep quality had a clear association with PSG variables thought to indicate good sleep, and this association was similar between men and women.

## Supplementary Material

Supplementary_files_zpag048
