# Supplementary material for: Gender differences in objective and self-reported sleep
Source: Sleep Adv. 2026 Apr 27;7(2):zpag048. doi: 10.1093/sleepadvances/zpag048 (PMC13198721; doi:10.1093/sleepadvances/zpag048)
Supplement: Supplementary_files_zpag048 [file supplementary_files_zpag048.docx]

**Gender differences in objective and subjective sleep**

Torbjörn Åkerstedt^1,2^, Johanna Schwarz^2,1^, Eva Lindberg^3^, Jenny Theorell-Haglöw^3^

1 Department of Clinical Neuroscience, Karolinska Institute, Stockholm, Sweden

2 Department of Psychology, Stockholm University, Stockholm, Sweden

3 Department of Medical Sciences, Respiratory, Allergy and Sleep research, Uppsala University, Sweden

[torbjorn.akersted@ki.se](mailto:torbjorn.akersted@ki.se), [johanna.schwarz@su.se](mailto:johanna.schwarz@su.se), eva.lindberg@medsci.uu.se jenny.theorell.haglow@medsci.uu.se

Correspondence should be addressed to: Professor Torbjörn Åkerstedt, Department of Clinical Neuroscience, Karolinska institute, 17177 Stockholm, Sweden.

[torbjorn.akerstedt@ki.se](mailto:torbjorn.akerstedt@ki.se)

Phone: +46 737078928;

**Table S1.** Mean±sd for gender groups.

|  | **Women**  **Mean±se** | **Men**  **Mean±se** |
| --- | --- | --- |
| **TST** | 414±4.5 | 396±4.0 |
| **Sleep efficiency (%)** | 89.6±0.7 | 86.4±0.7 |
| **WASO (min)** | 48.22±3.4 | 62.2±3.1 |
| **Sleep latency** | 12.9±1.3 | 11.3±1.2 |
| **N3 latency (min)** | 42.7±4.2 | 51.4±3.8 |
| **REM latency (min)** | 112.6±4.7 | 93.9±4.2 |
| **AHI (/h)** | 7.7±1.0 | 8.5±0.9 |
| **N1%** | 8.3±0.5 | 13.2±0.5 |
| **N2%** | 53.4±0.6 | 50.8±0.6 |
| **N3%** | 18.3±0.5 | 16.1±0.5 |
| **REM%** | 20.0±0.4 | 19.9±0.4 |
| **Awakenings/h** | 1.02±0.13 | 1.72±0.15 |
| **Time awake/awakening** | 8.18±0.6 | 6.38±0.6 |
| **Sleep quality index** | 3.52±0.1 | 4.00±0.1 |
|  |  |  |
| **Sleep duration subj/obj** | 0.96±0.1 | 0.99±0.1 |
| **Nr Awakenings subj/obj** | 0.63±0.1 | 0.28±0.1 |
| **Time awake subj/obj** | 1.27±0.2 | 1.32±0.2 |
| **Sleep latency, subj/obj** | 5.33±0.6 | 4.39±0.6 |

WASO=Time awake after sleep onset; TST=Total sleep time; REM=Rapid

eye movement sleep. AHI=Apnea-Hypopnea-Index.

**Table S2**. Mean±se for age groups for variables without a significant effect of gender or gender*age interaction in table 2.

|  | **Age 33-49 years**  **Women/men** | **Age 50-64 years**  **Women/men** | **Age65+ years**  **Women/men** |
| --- | --- | --- | --- |
| **Sleep latency** | 10.7±2.0/10.7±2.0 | 13.4±1.9\|11.2±1.9 | 18.9±2.1\|14.2±2.1 |
| **AHI (/h)** | 3.1±1.3/4.4±$1.3$ | 9.3±1.2/8.4±1.2 | 15.7±1.3\|15.4\|1.3 |
| **REM%** | 20.6±0.6/21.5±0.7 | 20.0±0.6/19.0±0.6 | 17.6±0.7/16.6±0.6 |
